# Mathematical modeling of the cardiovascular autonomic control in healthy subjects during a passive head-up tilt test

**DOI:** 10.1038/s41598-020-71532-7

**Published:** 2020-10-05

**Authors:** Yurii M. Ishbulatov, Anatoly S. Karavaev, Anton R. Kiselev, Margarita A. Simonyan, Mikhail D. Prokhorov, Vladimir I. Ponomarenko, Sergey A. Mironov, Vladimir I. Gridnev, Boris P. Bezruchko, Vladimir A. Shvartz

**Affiliations:** 1 Department of Innovative Cardiological Information Technology, Institute of Cardiological Research, Saratov State Medical University, Saratov, Russia; 2 Department of Surgical Treatment for Interactive Pathology, Bakulev Scientific Center for Cardiovascular Surgery, Moscow, Russia; 3 Laboratory of Nonlinear Dynamics Modeling, Saratov Branch of the Institute of Radio Engineering and Electronics of Russian Academy of Sciences, Saratov, Russia; 4 Department of Dynamic Modeling and Biomedical Engineering, Saratov State University, Saratov, Russia; 5 Department of Atherocslerosis and Chronic Ischemic Heart Disease, Institute of Cardiological Research, Saratov, Russia

**Keywords:** Computer modelling, Applied mathematics

## Abstract

A mathematical model is proposed for the autonomic control of cardiovascular system, which takes into account two separated self-exciting sympathetic control loops of heart rate and peripheral vascular tone. The control loops are represented by self-exciting time-delay systems and their tone depends on activity of the aortic, carotid, and lower-body baroreceptors. The model is used to study the dynamics of the adaptive processes that manifest in a healthy cardiovascular system during the passive head-up tilt test. Computer simulation provides continuous observation of the dynamics of the indexes and variables that cannot be measured in the direct experiment, including the noradrenaline concentration in vessel wall and heart muscle, tone of the sympathetic and parasympathetic control, peripheral vascular resistance, and blood pressure. In the supine and upright positions, we estimated the spectral characteristics of the model variables, especially in the low-frequency band, and the original index of total percent of phase synchronization between the low-frequency oscillations in heart rate and blood pressure signals. The model demonstrates good quantitative agreement with the dynamics of the experimentally observed indexes of cardiovascular system that were averaged for 50 healthy subjects.

## Introduction

Mathematical modeling is an important tool for investigation of human organism^1^ due to unsolvable problems with human experimental studies. Design of the experiments is limited by risk of causing harm to subject’s health^2^ and direct measurement of necessary physiological data is often restricted by technical and financial limitations. Interpretation of the results is also often complicated by high nonstationarity of biological systems^3,4^.

Additional complications arise with investigation of the complex control systems such as cardiovascular autonomic control^5^. In particular, heart is regulated by both sympathetic and parasympathetic control loops that exhibit dynamics on similar time scales (about 0.1 Hz). Dynamics of the two loops cannot be separated via spectral analysis and their individual input in the circulation control is hard to estimate^6,7^. This problem cannot be fully solved even by carrying out the active tests with the selective sympathetic or parasympathetic blockers such as propranolol, arfonad, and atropine, since the blockade of one regulatory loop inevitably leads to changes in the dynamics of another loop. Similar behavior manifests itself in experimental data from patients with the dysfunction of one of the systems, in particular, in patients with left ventricular assist device^8^.

Because of the aforementioned problems, studies in vivo are often complimented by the development of mathematical models^9–17^ and numerical simulation, which lead to significant progress in fundamental understanding of cardiovascular control. For example, the development of complex mathematical models from the first principles that simulate individual and collective dynamics of autonomic control loops can benefit the advancement of personalized medicine, computer simulation of various tests without a risk for the patient’s health, and approbation of new methods for the analysis of biological data. Possible applications include the modeling of the effects of arfonad administration^18^ and estimation of baroreflex sensitivity using the model construction from experimental data^19–21^. However, mathematical modeling is hindered by high complexity of cardiovascular system (CVS). Even if the model is limited to autonomic control of circulation, the simplification is necessary, which leads to a limited description of a specific pathology or effect. For example, a passive head-up tilt test^22–25^ is one of the most common tests to investigate autonomic control adaptation both in medical practice and fundamental studies. There are a number of cardiovascular models^26–29^ capable of precise simulation of arterial pressure (AP) and heart rate dynamics during a passive tilt test.

Aforementioned models do not consider the self-exciting nature of cardiovascular autonomic control. Objectives of the simulations were not affected by this simplification, but it made impossible to reproduce the effects that are associated with complex nonlinear dynamics of the control loops interaction. In particular, earlier we revealed the synchronization between the low-frequency (LF) oscillations (with a central frequency of about 0.1 Hz) in RR-intervals and photoplethysmogram (PPG) signals and proposed a method for its quantitative estimation^30^. It is known that PPG allows indirect estimation of the frequency properties of arterial pressure variability (APV), since PPG signal is influenced to a strong degree by oscillations of AP^31^. Also, we demonstrated a perspective clinical application of LF oscillation synchronization to the estimation of fatal cardiovascular risk after myocardial infarction^32,33^, and to the personalization of medical therapy for myocardial infarction patients^34^ and hypertensive patients^35^.

The existing models of CVS are capable of precise quantitative simulation of AP and heart rate dynamics during passive tilt test even for individual patients, but underling processes in autonomic control are simplified and cannot fully simulate the real system. For the fundamental study of CVS, it may be more advantageous to use a model that is not specifically tailored to simulation of the tilt test, but is developed from the first principles to make a detailed representation of the autonomic control.

For that purpose, we propose a modification of the well-established model of autonomic control of circulation^14^ with the self-exciting loops of heart rate and AP autonomic control. Earlier, we demonstrated the ability of the modified model to reproduce the statistical characteristics of CVS of healthy human under resting conditions and patients with hypertension. The introduction of the self-exciting control loops also allowed quantitative simulation of synchronization between the control loops and respiration with a linearly increasing frequency^36^ and simulation of chaotic dynamics of CVS^15^.

For modeling complex biological objects such as CVS, one of the three approaches is commonly used: (i) modeling the shape of the signals without taking into account the physiological meaning of the model coefficients^36^; (ii) model construction from the first principles with further adjustment of the model parameters to achieve better correspondence (on average) with experimental data^11,14,15^; (iii) model reconstruction including parameters directly from experimental data^37–40^. Our study is aimed to define the model structure and to fit the model parameters to the ensemble-averaged experimental data. The obtained results can be used as a starting point for techniques of model structure identification directly from real data^37–39,41^.

In this study, the adjusted model of the autonomic control is used for qualitative simulation of head-up tilt test. Dynamics of autonomic control signals during adaptation to orthostasis is studied to gain insight in physiology of real control system. To simplify the model, we do not take into account the microcirculation and humoral factors, the respiration is introduced in a simplified manner, and the hydrodynamics is reduced to a minimum.

## Material and methods

### Ethical approval

Design of this study was approved by the Ethics Committee of the Saratov State Medical University (Saratov, Russia) in 2017. Informed consent was obtained from all participants. All procedures performed in the studies involving human participants were in accordance with the ethical standards of the institutional research committee and with the 1964 Helsinki Declaration and its later amendments or comparable ethical standards.

### Description of base mathematical model of cardiovascular autonomic control

**Figure 1:**
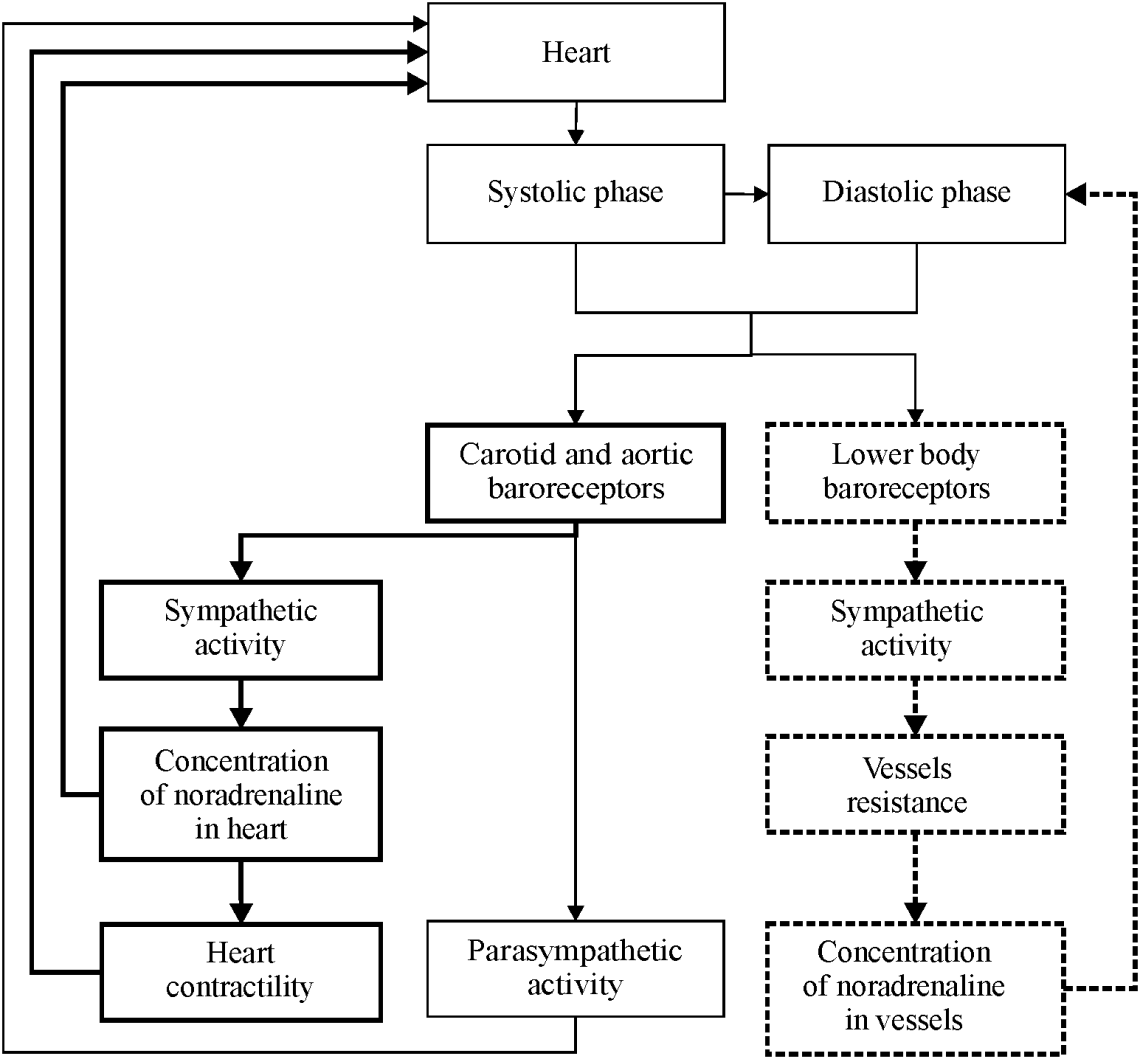
A scheme of the proposed model. Elements of the sympathetic regulation of heart rate and contractility are marked by bold lines. Elements of the autonomous control loop of vascular tone are marked by dashed lines.

Earlier, we proposed the modifications^15,18^ to the well-established mathematical model of a cardiovascular autonomic control^14^. In this study, we made further modifications and added the second autonomic control loop (Fig. 1).

Heart is represented by sinus node^14^. Heart rate is influenced by sympathetic and parasympathetic branches of autonomic nervous system. Sinus node activation leads to the systolic phase of the cardio cycle, during which aortic AP steeply rises. During this phase, the pressure depends on diastolic pressure in the previous cardio cycle, duration of the previous cardio cycle, and heart contractility. Heart contractility depends on the duration of the previous cardio cycle, concentration of noradrenalin in heart muscle, and vascular walls. To model respiration, we use a correlated stochastic process, which properties are estimated from experimental data.

The systolic phase of steep increase is followed by the diastolic phase, in which pressure slowly decreases due to blood runoff from the arterial windkessel. The decrease rate depends on elastic properties of aorta and resistance of peripheral vessels, which depend on mechanical properties and tone of the vessels. Peripheral vascular tone is influenced by the sympathetic activity.

Important feature of the model is two separated locations of the baroreceptors: aortic arch with carotid sinus and arteries of lower body, including internal organs. Signals from both baroreceptor groups are processed independently by centers of the autonomic control. The baroreceptors are sensitive to absolute value of AP and rate of its change, in accordance to Warner experimental results^42^. The baroreceptor output activates the sympathetic branch of the autonomic control that increases heart rate and tone of the lower-body arteries. Activation of baroreceptors also depresses the parasympathetic branch of autonomic control, which leads to further increase in heart rate^14,43^. To simulate passive transition from supine to upright position during a tilt test, we introduce additional pressure to the baroreceptors of lower body that reflects hydraulic pressure of blood.

To model autonomic control system, we do not use the simplified linear equation ^14,43^ and following Guild et al.^44^ employ the self-exciting equations that can exhibit stable 0.1 Hz oscillations, which can be observed in the spectra of the real AP and RR-interval signals. Activity of the sympathetic autonomic control loops of heart rate and contractility, and also of arterial vessel tone, lead to changes in concentration of noradrenaline, respectively in heart and vessels. Concentration of noradrenaline reacts to changes in activity of the sympathetic control loops with a delay, which is caused by finite speed of nervous transition and chemical reactions^14,43^.

Changes in the concentration of noradrenaline in heart muscle and changes in the activity of the parasympathetic branch are defining the corresponding changes in the sympathetic and parasympathetic influence on heart rate. No separate equation represents concentration of acetylcholine, since the production and decay of this hormone proceeds with higher rate in comparison to other modeled processes^14,43,45^. For the initial estimation of the majority of parameters, we used the existing models^13,14,43^ and the results of our previous studies^15,19,39^.

### Experimental data

To verify and adjust the parameters of the proposed model, we compare the model and experimental signals. Electrocardiogram (ECG), PPG from a middle finger of a right hand, PPG from an earlobe, and respiratory signals were simultaneously recorded for 10 min in 50 healthy subjects (25 men and 25 women aged 20–40 years and with average level of physical activity) at each stage of the head-up tilt test. At the end of each stage, we used noninvasive techniques to measure systolic arterial pressure (SAD) and diastolic arterial pressure (DAD). All subjects were fasting before the experiment. Recording sessions took place in the afternoon under spontaneous breathing. The recordings of respiration were used to control evenness of breathing.

We excluded from the analysis the series with forced inspiration and delays in breathing. For further analysis, only records without artifacts, extrasystoles, and considerable trends were used.

The head-up tilt test protocol includes the following stages:

(i) In the preliminary stage lasting 10 min, the subjects were lying in the horizontal position without signal recording.
(ii) 10-min signals were recorded in the horizontal resting position.
(iii) The subjects passively transitioned into the vertical position with a tilt angle of about 80°. To exclude transient processes, no signals were recorded in the next 5 min.
(iv) 10-min signals were recorded in the vertical position.

All experimental signals were recorded using the standard electroencephalograph analyzer EEGA-21/26 ‘Encephalan-131-03′ (Medicom MTD Ltd, Taganrog, Russia). The signals were recorded in a quiet room with controlled temperature. All signals were sampled at 250 sps and digitized at 14 bits.

### Data analysis

To compare the model and experimental data, we calculate the mean values and standard deviations for the following indexes: heart rate, systolic blood pressure, diastolic blood pressure, spectral, and synchronization indexes that are discussed further in more details. The indexes were calculated before and after passive transition from the supine to upright position.

The spectral indexes include the spectral power density of RR-interval signals in two frequency bands: LF index characterizes the spectral power density integrated in 0.04–0.15 Hz band and measured in ms^2^, and HF index characterizes the spectral power density integrated in 0.15–0.4 Hz band and measured in ms^2^. Calculations were carried out according to the methodological recommendations^46^.

Important feature of the modified model is the ability to simulate the frequency synchronization between the loops of autonomic control of heart rate and arterial vessel tone. To measure the strength of this synchronization, the total percent of phase synchronization (*S* index) was introduced in Ref.^30^. This index is calculated for a pair of signals: PPG (AP in the model) and RR-intervals. Calculation of *S* index consists of several steps: PPG (AP in the model) and RR-interval signals are filtered with 0.05–0.15 Hz band pass filter; oscillation phases are calculated from filtered signals; the difference between the obtained phases is calculated; automated algorithm^30^ is applied to detect horizontal sections of the phase difference signal; the horizontal sections correspond with intervals of frequency synchronization between the signals; and the total duration of the horizontal sections is calculated and divided by the total duration of the signals to estimate *S* index.

To estimate the dynamics of real CVS during a passive head-up tilt test and to study adaptation process in upright position, we also calculate the mean values and standard deviations for all model variables. These signals cannot be directly measured in experimental study on humans due to ethical and technical limitations.

### Model fitting and simulation

The model has 47 parameters, 14 of which have a weak influence on the minimization of the objective function (the function of ensemble-averaged HR, SAP, DAP, LF, HF indexes) and are taken as constant from the known models. Two parameters are estimated directly from experimental data. These parameters are the respiratory frequency *f*_*br*_ and standard deviation σ^2^(*ξ*) of the zero-mean Gaussian noise *ξ*, which is added to *f*_*br*_after each cycle of respiration. The $p_{hst}^{upright}$ coefficient is zero in the supine position.

In all, 31 parameters are adjusted during the fitting procedure, which is conducted using the gradient descend algorithm with a variable step. The objective function is chosen as follows:

1 $$ \begin{aligned} L({\vec{\mathbf{p}}}) & = \frac{{\left( {\overline{HR}_{M} ({\vec{\mathbf{p}}}) - \overline{HR} } \right)^{2} }}{{\overline{HR}^{2} }} + \frac{{\left( {\overline{SAP}_{M} ({\vec{\mathbf{p}}}) - \overline{SAP} } \right)^{2} }}{{\overline{SAP}^{2} }} + \frac{{\left( {\overline{DAP}_{M} ({\vec{\mathbf{p}}}) - \overline{DAP} } \right)^{2} }}{{\overline{DAP}^{2} }} \\ & \quad + \frac{{\left( {\overline{LF}_{M} ({\vec{\mathbf{p}}}) - \overline{LF} } \right)^{2} }}{{\overline{LF}^{2} }} + \frac{{\left( {\overline{HF}_{M} ({\vec{\mathbf{p}}}) - \overline{HF} } \right)^{2} }}{{\overline{HF}^{2} }}, \\ \end{aligned} $$

where ${\vec{\mathbf{p}}}$ is the vector of coefficients being fitted, $\overline{HR}_{M} ({\vec{\mathbf{p}}})$, $\overline{SAP}_{M} ({\vec{\mathbf{p}}})$, $\overline{DAP}_{M} ({\vec{\mathbf{p}}})$, $\overline{LF}_{M} ({\vec{\mathbf{p}}})$, and $\overline{HF}_{M} ({\vec{\mathbf{p}}})$ are the values of model HR, SAP, DAP, LF, and HF indexes, respectively, averaged for 50 realizations of the model, and $\overline{HR}$, $\overline{SAP}$, $\overline{DAP}$, $\overline{LF}$, and $\overline{HF}$ are the averaged indexes estimated from 50 healthy subjects. Each term in (1) is divided by the ensemble-averaged value of the corresponding parameter. Thus, all parameters are of the same order. The fitting process is finished when

2 $$ L({\vec{\mathbf{p}}}) < \varepsilon . $$

We empirically estimated the value of *ε* as 0.05. For such *ε*, the inequality (2) is satisfied when the difference between the model and experimental indexes is less than 10%.

The parameters are fitted for the upright position and the obtained values are used in the free run of the model. The first 120 min are missed from every signal to exclude the transient process. The model equations and the initial and fitted values of parameters are presented in the Supplementary Information file.

## Results

**Figure 2:**
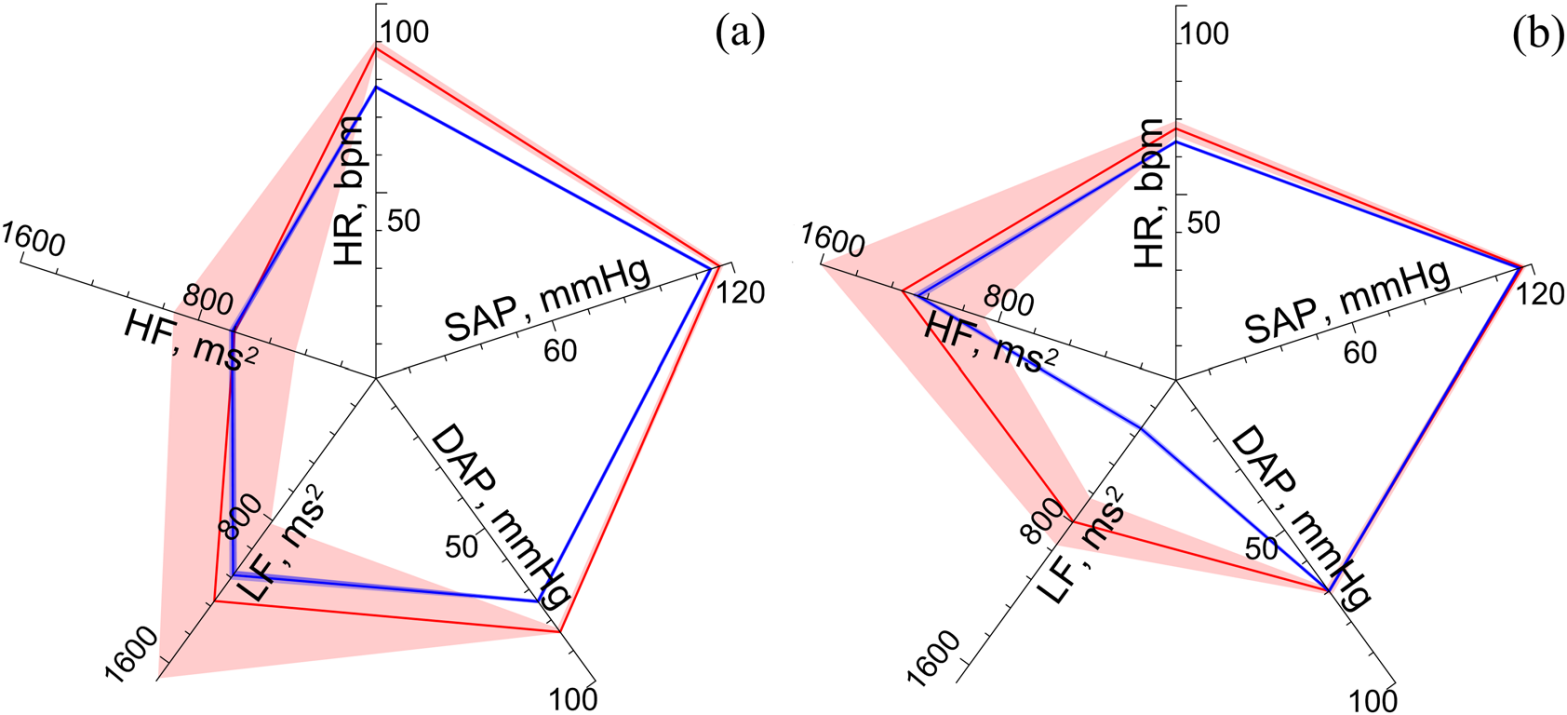
Comparison of the experimental and model indexes in the upright and supine positions. (**a**) Radar chart, that illustrates the fitting of the HR, SAP, DAP, LF, and HF model indexes in the upright position; (**b) ** modeling of the healthy subject in the supine position in the free run mode ($p(t)_{hst} = 0$). Mean values of the experimental and model indexes are shown with red and blue lines, respectively. Standard deviations of the experimental and model indexes are shown with pink and light blue, respectively.

After the minimization, the objective function (1) takes the value $L({\vec{\mathbf{p}}}) = 0.042$. Figure 2(a) illustrates the merit and goodness of the parameter fitting in the upright position. The largest relative error of fitting is observed for $\overline{DAP}_{M} ({\vec{\mathbf{p}}})$ and takes the value of 11.9%. The minimal relative error of fitting is observed for $\overline{HF}_{M} ({\vec{\mathbf{p}}})$ and takes the value of 1.7%. Figure 2(b) shows the model indexes obtained for the supine position ($\alpha (t) = 0$) with the model parameters fitted for the upright position. Index $\overline{LF}_{M} ({\vec{\mathbf{p}}})$ is smaller than the corresponding experimental index, but the indexes $\overline{HR}_{M} ({\vec{\mathbf{p}}})$, $\overline{SAP}_{M} ({\vec{\mathbf{p}}})$, and $\overline{DAP}_{M} ({\vec{\mathbf{p}}})$ demonstrate even better correspondence to the experimental data than in the supine position.

**Figure 3:**
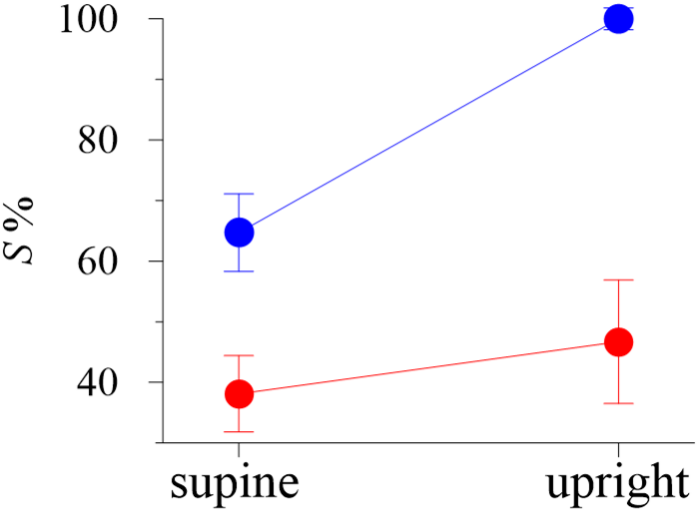
Ensemble-averaged values of the total percent of phase synchronization index in the supine and upright positions. Experimental data are shown with red dots. Model data are shown with blue dots. The whiskers represent the standard deviation.

From the model and experimental data, we estimated the total percent of phase synchronization *S* and plotted it in Fig. 3. In the upright position, the *S* index is higher than in the supine position both in the model and in the real system that correlates with the result of Ref.^47^. However, the experimental *S* values are smaller than the model ones.

We obtained the following model variables from each realization: ECG signals, signals of the baroreceptor activity in carotid sinus and lower body arteries (*v*_*b*_(*t*) and $v_{b}^{l} (t)$), activity of the heart rate sympathetic control (*v*_*s*_(*t*)), heart contractility (*s*(*t*)), sympathetic control of arterial vessel tone ($v_{s}^{l}$(*t*)), activity of the parasympathetic control (*v*_*p*_(*t*)), noradrenaline concentration in heart muscle (*c*_*c*_(*t*)), noradrenaline concentration in vessel wall (*c*_*v*_(*t*)), factors of sympathetic and parasympathetic heart rate control (*f*_*s*_(*t*) and *f*_*p*_(*t*)), SAP, DAP, and total resistance of the peripheral vessels (*R*(*t*)).

Table 1 shows the mean values of model signals in the supine and upright positions after the transient process. These values cannot be directly experimentally measured in humans.

**Table 1:** Mean values of variables in the model of the cardiovascular autonomic control during a tilt test.

Variable	Supine position	Upright position
*R*(*t*)	0.80	0.77
*s*(*t*)	46.0	37.7
*v*_*b*_(*t*)	5.25	3.22
$v_{b}^{l}$(*t*)	5.25	7.05
$v_{s}^{l}$(*t*)	2.02	0.55
*v*_*s*_(*t*)	1.98	3.02
*v*_*p*_(*t*)	1.05	0.65
*c*_*c*_(*t*)	0.14	0.21
*c*_*v*_(*t*)	2.23	0.75
*f*_*s*_(*t*)	1.43	1.67
*f*_*p*_(*t*)	0.90	0.93

The results presented in Fig. 2 and Table 1 count in favor of a hypothesis that complex adaptation processes take place in the circulatory system during a passive transition from the supine to upright position.

**Figure 4:**
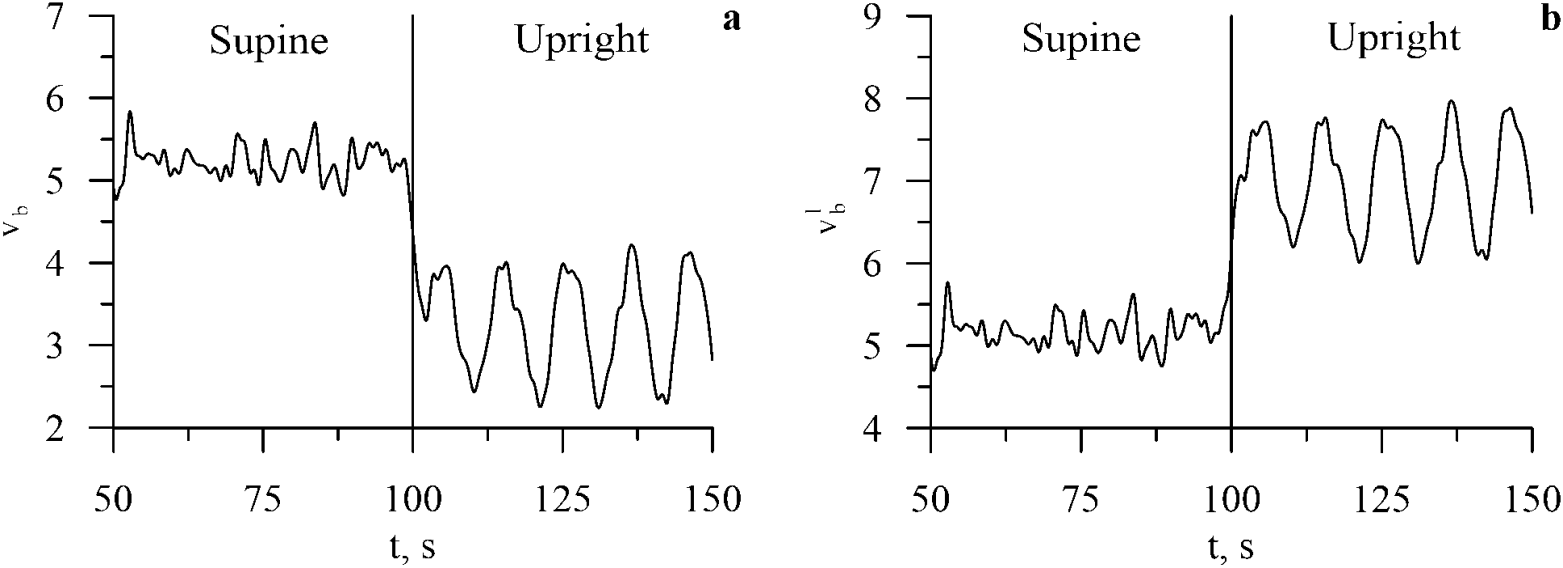
Dynamics of the model heart rate (HR) and blood pressure (BP) variables during the transition from the supine to the upright position.

**Figure 5:**
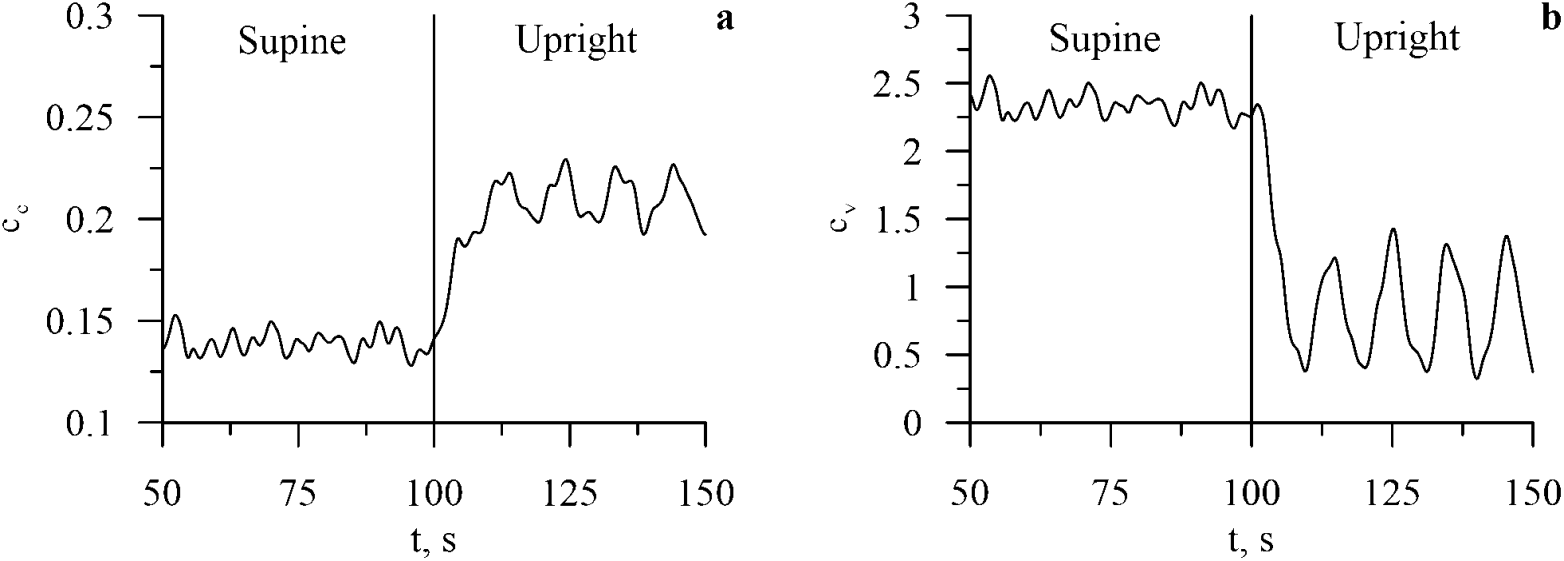
Dynamics of the carotid baroreceptors (*v*_*b*_(*t*)) and the lower-body arteries baroreceptors ($v_{b}^{l}$(*t*)) during the transition from the supine to upright position.

Blood outflows from upper body during a transition to the supine position leads to a decreased baroreceptor activity (*v*_*b*_(*t*)) in upper body and increased baroreceptor activity in lower body arteries ($v_{b}^{l}$(*t*)) (Fig. 4). Changes in the baroreceptor activity lead to changes in the sympathetic activity. Sympathetic influence on heart rate rises (*f*_*s*_(*t*) in Table 1) and noradrenaline concentration in heart muscle rises (*c*_*c*_(*t*)) (Fig. 5) that negates increased parasympathetic influence on heart rate. Sympathetic activation also leads to increase in spectral power in the LF band of RR-interval spectrum, which is also present in the experimental data (see Fig. 2).

**Figure 6:**
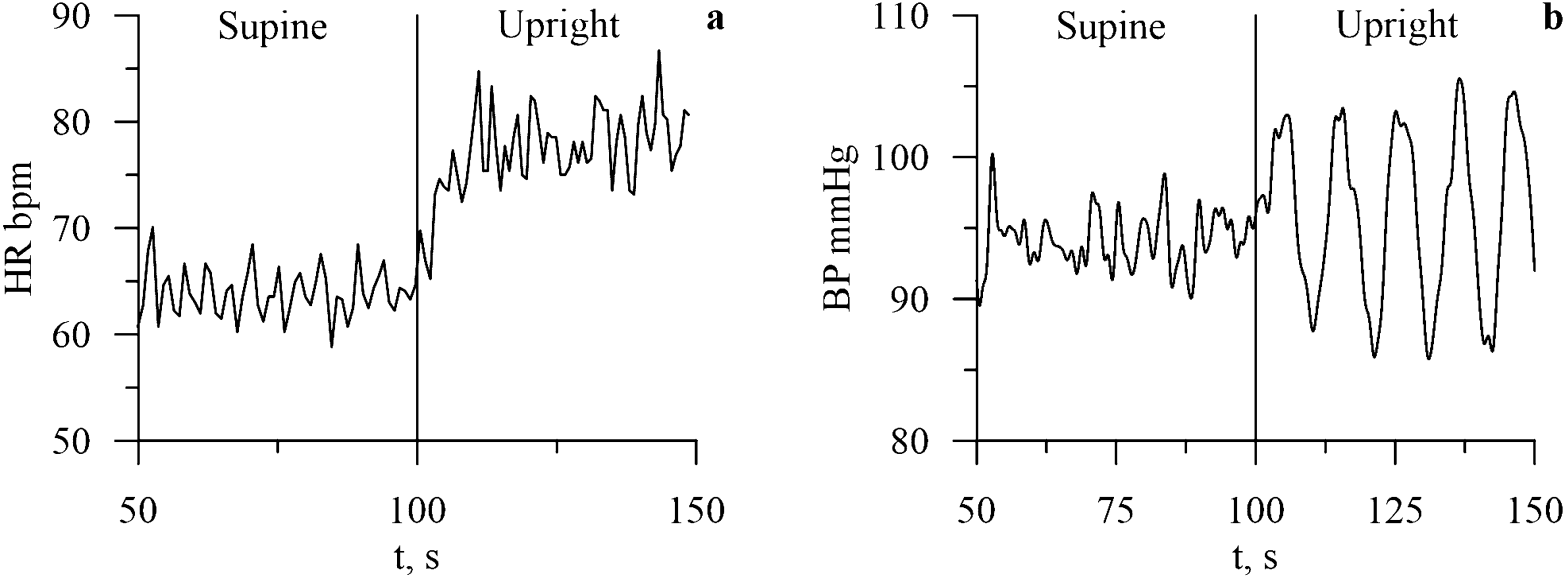
Dynamics of the noradrenaline concentration in heart (*c*_*c*_(*t*)) and vessels (*c*_*v*_(*t*)) during the transition from the supine to upright position.

Increase in the RR-interval LF power is explained by the stronger 0.1 Hz self-oscillations, (see Fig. 6). Stronger self-oscillations lead to stronger synchronization (increased *S* index) between subsystems of the autonomic control in LF band (see Fig. 2). Another observed effect is the rising of AP in lower body, which leads to increased sympathetic control of the vessels ($v_{s}^{l}$(*t*) and *c*_*v*_(*t*)) (Table 1) causing a decrease in vessel resistance *R*(*t*).

Thus, CVS transits into a new steady state with, at average, higher activity of the sympathetic control, higher heart rate, AP, and power of RR-intervals in the LF band. Increase in the sympathetic activity is partly compensated by lower heart contractility (*s*(*t*)), which helps containing the pressure within the physiological limits.

## Discussion

In the present paper, the mathematical model for the autonomic control of CVS has been proposed from the first principles. The proposed model takes into account the modern understanding of the self-exciting nature of the autonomic control of heart rate and heart contractility. Autonomic control loop in the earlier model took into account only the influence of the baroreceptors from the main reflexogenic zones (aortic and sinocarotid). However, some experimental results^44^ suggest the functional independence of the LF oscillations in RR-intervals and PPG. Earlier, we also observed the phenomenon of the synchronization between the LF oscillations in RR-intervals and PPG^30,33,48^. Typically, the intervals of synchronization are alternating with the intervals of the desynchronized behavior. We hypothesize that such complex dynamics is caused by coupled activity of the sympathetic control loops from aortic and sinocarotid receptor zones (regulation of heart rate) and independent receptors of peripheral arteries of lower body (regulation of PPG).

Before implementing the second control loop into the model, we searched for reported experimental evidence of pressure sensitive receptors in the lower body. Many experimental results suggest the existence of such receptors^49^, but there are no model or functional diagrams that consider their self-exciting nature. Therefore, to model the receptors of the second control loop, we used the same equations that were used for modeling the baroreceptors.

Complex dynamics of two coupled negative feedback loops led to overall positive reaction of the heart rate and AP to the increase of the blood pressure in the lower body, caused by the orthostatic stress. May be such coupled dynamics could explain the increase of heart rate and AP during stretch of the thoracic aorta reported in Ref.^50^.

The model structure reflects the modern understanding of the autonomic control of CVS, and its parameters have a physiological meaning. The proposed model was used to study the dynamics of the autonomic control of circulation in healthy subjects during the passive head-up tilt test. The passive head-up tilt test is known to cause steep increase in volume of blood in lower-body vessels, which leads to the decrease in venous return and stroke volume, and also to activation of the autonomic control loops, including aortic baroreceptors, cardiopulmonary baroreceptors, sympathetic control of heart rate, and peripheral vessel tone. These factors lead to the increase in heart rate and diastolic AP^51,52^, while systolic AP stays practically unchanged^52^.

In contrast to other models^14,53^, the modified model contains two self-exciting loops of autonomic control that are connected to the baroreceptors of aorta and lower-body arteries. Introduction of the second control loop is aimed to estimate the influence of baroreceptors of lower-body arteries on systemic circulation during a tilt test. The modified model is able to simulate average dynamics of the CVS indexes in healthy subjects during a tilt test. In accordance with the results of other authors^54–58^, SAP stayed practically unchanged. DAP and heart rate become higher. However, the proposed model demonstrate significantly worse simulation of individual dynamics of the aforementioned indexes that is most likely due to a high variability in parameters of autonomic control in healthy subjects. It also explains the smaller standard deviation of the model indexes in relation to the experimental data (Fig. 2). We believe that our model is able to reproduce the general basic characteristics of autonomic control of circulation that are typical for healthy subjects, but it did not take into account their individual features.

The modified model adopts a number of simplifications that revolve around the idea of modeling two independent self-exciting loops of autonomic control, which are driven by separated groups of receptors. When introducing a second control loop, we tried to achieve the full independence of the LF oscillations in RR-intervals and PPG. Therefore, we made the lower-body sympathetic control loop to be solely responsible for control of total peripheral resistance (TPR), while the sinocarotid and aortic baroreflex was made solely responsible for control of the heart rate and contractility. We also introduced the integral index *R*(*t*) for TPR instead of hydrostatical model of vascular bed. Such rigid specialization of the control loops made model simpler, but also led to the incorrect simulation of the TPR, which was represented as an integral index *R*(*t*). The transition to the upright position caused the blood pressure to rise in the lower body, and the lower-body control loop tried to compensate for it by decreasing the *R*(*t*) by 3.7%, while in a human body, the TPR should increase^24,59^ to sustain the optimal brain circulation.

The upper-body control loop in our model feels the drop in the AP, but cannot directly compensate it, since this loop regulates in the model only the heart rate and heart contractility *s*(*t*), but not the TPR. Such reaction to a tilt test is more typical for the patients with vegetative dysfunction and leads to the syncope. This problem can be solved by giving the aortal/sinocarotid baroreflex loop the direct control over the TPR, so that the overall control of TPR will be ensured by both loops. Another important modification to be added to the future model is a more detailed representation of the hydrostatical processes.

However, the present model still shows good correspondence with the experimental data obtained from the subjects in the upright position and agrees well with the established model^60^. Addition of nonlinear loops of autonomic controls allowed us to model the SAP dynamics better than the model^60^, since in our model, SAP decreases after the transition to the upright position, while in the model^60^, it slightly increases.

In our study, the respiration is modeled as a stochastic process, which statistical properties are estimated from the experimental data (see the Supplementary Information file). However, with such approach, we managed to achieve the best fit (in relation to other indexes of the model) of the $\overline{HR}_{M} ({\vec{\mathbf{p}}})$ index in the upright position (Fig. 2). During the free run modeling in the supine position, this index is slightly smaller than the corresponding experimental value. However, $\overline{LF}_{M} ({\vec{\mathbf{p}}})$ in the free run is significantly lower than the corresponding experimental value. It can be due to the simplifications of the model, since we use the constant amplitude of respiration that leads to underestimation of the respiratory influence on the sympathetic control (see the Supplementary Information file).

Better representation of the LF dynamics can be achieved with more detailed modeling of respiration that takes into account the feedback mechanisms^14,61^ or through the use of experimental recordings of respiration as in Ref.^62^. Simplification of the respiration description in the model also leads to the increase of *S* index (Fig. 3), since the respiration is important for the RR-interval and AP coupling^62^. Such results highlight the importance of respiration, but more detailed modeling is limited due to the lack of a priory information. Many studies propose different models of respiratory influence on CVS^14,43,61,62^. We also did not take into account the well-known effect of cardiopulmonary reflex on control of the systemic blood pressure. Similar simplification was made in Ref.^45^, where the model had good agreement with the ensemble-average experimental data.

The model showed that increase in the synchronization between baroreflectory loops can help balancing local baroreflectory effects on the level of the heart and peripheral vessels in order to sustain systemic AP during a transition to the upright position. Such balance is achieved in the model without involvement of central mechanisms of the vascular tone control, which emphasizes a potential importance of the segmental circulatory sympathetic control. It is possible that such distributed excitatory effects can be even more enhanced and better balanced if combined with the vasomotion synchronization effects reported in a number of studies^63,64^.

Because of high complexity of CVS, we had to adopt a number of limitations when modeling the cardiovascular autonomic control. Our model does not take into account the humoral regulation and other processes with time scales of more than 20 s and the local intracardial control mechanisms: the Bowditch effect, the Bayleys effect, etc. Moreover, the control of vascular tone includes not only local reflex mechanisms modulating sympathetic activity, but also central input that is not included in the present model. This hypothesis is supported by the other studies^65–67^, which reveal an interaction between the higher nervous activity and the autonomic control. The model does not consider the oscillations in microcirculation, which are caused by the myogenetic and neurogenic processes and have time scales similar to those of the autonomic control, and also the influence from the control of systemic circulation^63,64,68^. No separate equation represents the concentration of acetylcholine, since the production and decay of this hormone proceeds with higher rate in comparison to other modeled processes. Similar simplified approach to modeling of parasympathetic control was adopted in Ref^45^. and it had no significant negative effect on the model ability to simulate group-averaged experimental data. We also adopted the limitations and simplifications that are discussed in details in the studies of other authors^14,43^.

We believe that disruption of coupling between the autonomic control loops that related to the baroreceptors in aorta and lower-body arteries can take part in pathogenesis of the orthostatic hypotension. It is known that lowered baroreceptor sensitivity is one of pathogenic factors of the orthostatic hypotension^66^. It commonly refers to the sensitivity of the aortic and carotid baroreceptors. However, changes in biophysical characteristics of other arterial baroreceptors can also influence the development of hypotension.

## Conclusion

We have proposed the modification to the model of cardiovascular autonomic control^14^ that includes the use of two self-exciting loops of the autonomic control, which are related to the baroreceptors in aorta and lower-body vessels. The importance of the lower-body baroreceptors is shown during a passive transition to the upright position in establishing of the stable condition of CVS. Established stable condition has higher mean arterial pressure, heart rate, and sympathetic tone, and lower parasympathetic tone and heart contractility. Stronger low-frequency oscillations are observed in the signals taken directly from sympathetic control loop of heart rate and tone of lower-body vessels. This finding can explain the increase of spectral power density in the low-frequency band that is typical for healthy subjects after a transition to the upright position during tilt test.

## Supplementary information

Supplementary file1
