# Supplementary material for: Mathematical modeling of the cardiovascular autonomic control in healthy subjects during a passive head-up tilt test
Source: Sci Rep. 2020 Oct 5;10:16525. doi: 10.1038/s41598-020-71532-7 (PMC7536219; doi:10.1038/s41598-020-71532-7)
Supplement: Supplementary file 1 — Supplementary file1 [file 41598_2020_71532_MOESM1_ESM.doc]

**Supplementary information** for the article

**Mathematical modeling of the cardiovascular autonomic control in healthy subjects during a passive head-up tilt test**

*Authors:* Ishbulatov Yu.M.^1,2^, Karavaev A.S.^1,3,4^, Kiselev A.R.^1,2,4^, Simonyan M.A.^5^, Prokhorov M.D.^3^, Ponomarenko V.I. ^3,4^, Mironov S.A.^1^, Gridnev V.I.^1,4^, Bezruchko B.P.^3,4^, Shvartz V.A.^2^

*Organizations:*

^1^ Department of Innovative Cardiological Information Technology, Institute of Cardiological Research, Saratov State Medical University, Saratov, Russia

^2^ Department of Surgical Treatment for Interactive Pathology, Bakulev Scientific Center for Cardiovascular Surgery, Moscow, Russia

^3^ Laboratory of Nonlinear Dynamics Modeling, Saratov Branch of the Institute of Radio Engineering and Electronics of Russian Academy of Sciences, Saratov, Russia

^4^ Department of Dynamic Modeling and Biomedical Engineering, Saratov State University, Saratov, Russia

^5^ Department of Atherocslerosis and Chronic Ischemic Heart Disease, Institute of Cardiological Research, Saratov, Russia

**Description of the model equations**

The model contains four first-order differential equations with delay. Heart rate is set by integrate-and fire model taken from Refs. [1, 2], but originally proposed in Ref. [3]:

, (1)

where is the phase of the heart contraction, *T*_0_ is the period of the denervated heart contractions, $f_{s}(t)$ and $f_{p}(t)$ are sympathetic and parasympathetic factors, and ξ(*t*) is a red noise with normal distribution and 1/*f* spectrum.

Heart contraction initiates the systolic phase of the cardiac cycle, in which blood pressure is described as in Refs. [1, 2]:

$p_{sys}(t)=D_{i-1}+s(t)\frac{(t-T_{i-1})}{T_{sys}}\exp\left(1-\frac{t-T_{i-1}}{T_{sys}}\right)+k_{p}^{B}B(t)$, (2)

where $D_{i-1}$ is the diastolic pressure in the previous cycle, $T_{i-1}$ is the duration of the previous cycle, $T_{sys}$ is the fixed duration of a systole, *s*(*t*) is the heart contractility, *k_p_^B^* is a nondimentional coefficient, and $B(t)$ is the respiration modeled with a sinusoidal signal, which frequency is slightly varied each respiratory cycle. After the full period of the sinusoidal signal, a zero-mean normally distributed noise $\zeta_{j}$ is added to the frequency of the respiration *f_br_*: $B(t)=\sin\left(2\pi\left(f_{br}+\zeta_{j}\right)t\right)$. The heart contractility [1, 2] is described as follows:

$s(t)=s'(t)+\left(\hat{s}-s'(t)\right)\frac{s'(t)^{n_{c}}}{\hat{s}^{n_{c}}+s'(t)^{n_{c}}}$, (3)

where $s'(t)=s_{0}+k_{S}^{c}c_{c}(t)+k_{S}^{v}c_{v}(t)+k_{S}^{t}T_{i-1}$ and depends on the noradrenalin concentration in cardiac muscle $c_{c}(t)$ and vessel wall $c_{v}(t)$, and $\hat{s}$, $n_{c}$, $s_{0}$, $k_{S}^{c}$, $k_{S}^{v}$, and $k_{S}^{t}$ are coefficients.

Pressure $p_{dia}(t)$ in the diastolic phase is described by the following equation:

$\frac{dp_{dia}(t)}{dt}=-\frac{p_{dia}(t)}{R(t)C}$, (4)

where *C* is a constant characterizing the elastic properties of aorta and *R*(*t*) is the total peripheral resistance that depends on mechanical properties and tone of the arterial vessels:

$R(t)=R_{0}\left(1+k_{R}^{v}c_{v}(t)\right)$, (5)

where *R*_0_ is the resistance of vessels under resting conditions, *c_v_*(*t*) is the noradrenalin concentration in a vascular wall, and $k_{R}^{v}$ is a coefficient.

Blood pressure$p(t)$ is a combination of equations (2) and (4) for the current *i*th cardiac cycle:

$\left[\begin{matrix} p(t)=p_{sys}(t), t_{i}\leq t<t_{i}+T_{sys}, \\ p(t)=p_{dia}(t), t_{i}+T_{sys}\leq t<t_{i+1}. \end{matrix}\right.$ (6)

The baroreceptor reactions to changes in blood pressure are modeled as in Ref. [4]:

$v_{b}=k_{1}\left(p(t)-p_{0}-\frac{p_{hdr}}{3}\right)+k_{2}\frac{dp(t)}{dt},$ (7)

$v_{b}^{l}=k_{1}^{l}\left(p(t)-p_{0}^{l}+p_{hdr}\right)+k_{2}^{l}\frac{dp(t)}{dt}$, (8)

where $p_{0}$ and $p_{0}^{l}$ are the minimal levels of blood pressure recognized by the baroreceptors, $k_{1}$, $k_{2}$, $k_{1}^{l}$, and $k_{2}^{l}$ are coefficients, and $p_{hdr}$ are the changes in blood pressure caused by redistribution of blood due to the transition from the supine to upright position:

$p_{hdr}(t)=p_{hdr}^{upright}\sin\left(\alpha(t)\right)$, (9)

where $\alpha(t)$ is the angle of the body in relation to the horizontal plane. In the supine position, $p_{hdr}(t)=$0, and in the upright position, $p_{hdr}(t)=p_{hdr}^{upright}$. $\alpha(t)$ is described as follows:

$\left[\begin{matrix} \alpha(t)=0, t<T_{start}^{tilt}, \\ \alpha(t)=\frac{\pi}{8}t, T_{start}^{tilt}\leq t<T_{finish}^{tilt}, \\ \alpha(t)=\frac{\pi}{2}, t>T_{finish}^{tilt}, \end{matrix}\right.$ (10)

where $T_{start}^{tilt}$ is a beginning of the transition from the supine to upright position. This transition takes 4 seconds and $T_{finish}^{tilt}$ is the end time of the transition.

Activity of the carotid and lower-body baroreceptors influences the activity $v_{s}(t)$ and $v_{s}^{l}(t)$ of the separated loops of the sympathetic control:

$v_{s}(t)=a_{s}\tanh\left(b_{s}\left(v_{b}(t)\right)-v_{s}^{0}\right)+y_{s}+k_{s}^{r}B(t)$, (11)

$v_{s}^{l}(t)=a_{s}^{l}\tanh\left(b_{s}^{l}\left(v_{b}^{l}(t)\right)-v_{s}^{l0}\right)+y_{s}^{l}+k_{s}^{lr}B(t)$, (12)

where $v_{s}^{0}$ and $v_{s}^{l0}$ are the resting tones of sympathetic controls of heart rate and vessel tone, $a_{s}$, $b_{s}$, $y_{s}$, $k_{s}^{r}$, $a_{s}^{l}$, $b_{s}^{l}$, $y_{s}^{l}$, and $k_{s}^{lr}$ are coefficients. Equations (11) and (12) are similar to the equations of the self-exciting model of autonomic control proposed in Ref. [5]. The parasympathetic control loop activity *v_p_*(*t*) is associated with the carotid baroreceptors [1, 2]:

$v_{p}(t)=\max\left(0,v_{p}^{0}+k_{p}^{b}v_{b}(t)+k_{p}^{r}\left|B(t)\right|\right)$, (13)

where $v_{p}^{0}$ is the resting tone of the parasympathetic control and $k_{p}^{r}$ is a coefficient.

Changes of the sympathetic control of the heart rate and vascular tone $v_{s}(t)$ and $v_{s}^{l}(t)$ lead to changes in the noradrenalin concentration in heart and vessels, respectively:

$\frac{dc_{c}(t)}{dt}=-\frac{c_{c}(t)}{\tau_{c}}+k_{c}^{S}v_{s}\left(t-\theta_{c}\right)$, (14)

$\frac{dc_{v}(t)}{dt}=-\frac{c_{v}(t)}{\tau_{v}}+k_{v}^{S}\left(v_{S}^{l}\left(t-\theta_{v}\right)+k_{v}\right)$, (15)

where $c_{c}(t)$ and $c_{v}(t)$ are the noradrenalin concentrations in heart and vessel walls, $\tau_{c}$ and $\tau_{v}$ are relaxation times, $k_{c}^{S}$, $k_{v}^{S}$, and $k_{v}$ are coefficients, and $\theta_{c}$ and $\theta_{v}$ are the time delays caused by the time that noradrenalin needs to produce an effect on heart and vessel tone.

The sympathetic and parasympathetic control of baroreflectory regulation of heart rate is introduced as sympathetic factor [1, 2]:

(16)

and parasympathetic factor:

, (17)

where $\theta_{p}$ is the delay time, $k_{\phi}^{c}$, $k_{\phi}^{c}$, $\hat{c}_{c}$, $\hat{v}_{p}$, *n_s_*, and *n_p_* are coefficients, and *n_s_* and *n_p_* define the steepness of the transfer functions.

Sympathetic nervous system influences heart rate through changes in the noradrenalin concentration $c_{c}(t)$ that also influences heart contractility (3). Changes in the acetylcholin concentration (parasympathetic neurotransmitter) are significantly faster. This process is represented in $f_{p}(t)$ only as the time delay $\theta_{p}=0.5$ s.

Phase effectiveness curve [1, 2]:

(18)

takes into account the influence of phase of cardiac cycle on parasympathetic control loop.

The model parameters are listed below in the Supplementary Table S1.

**Parameters of the model**

Supplementary Table S1. Parameters of the proposed mathematical model of the cardiac autonomic control

| 1 | 2 | 3 | 4 | 5 | 6 |
| --- | --- | --- | --- | --- | --- |
| **#** | **Parameter** | **Initial value** | **Adjusted value** | **Physiological meaning** | **Source** |
| **The parameter values are taken from the other articles or estimated from experimental data or from physical considerations** | | | | | |
| 1 | $T_{sys}$ | 0.125 s | ‑ | Constant duration of systolic phase of heart contraction | [2] |
| 2 | $n_{c}$ | 2.5 | ‑ | ‑ | [2] |
| 3 | $s_{0}$ | -13.8 ml | ‑ | ‑ | [2] |
| 4 | $k_{S}^{c}$ | 10 mmHg | ‑ | ‑ | [2] |
| 5 | $k_{S}^{v}$ | 20 mmHg | ‑ | ‑ | [2] |
| 6 | $k_{S}^{t}$ | 45 mmHg·s^-1^ | ‑ | ‑ | [2] |
| 7 | $y_{s}$ | 1.0 | ‑ | ‑ | [5] |
| 8 | $\tau_{c}$ | 2.0 s | ‑ | Delay due to slow noradreniline [secretion](https://www.multitran.com/m.exe?s=secretion&l1=1&l2=2) | [2] |
| 9 | $\tau_{v}$ | 2.0 s | ‑ | Delay due to slow noradreniline [secretion](https://www.multitran.com/m.exe?s=secretion&l1=1&l2=2) | [2] |
| 10 | $k_{v}$ | 0.2 | ‑ | ‑ | [2] |
| 11 | $\hat{c}_{c}$ | 2.0 | ‑ | ‑ | [2] |
| 12 | $n_{s}$ | 2.0 | ‑ | ‑ | [2] |
| 13 | $\hat{v}_{p}$ | 2.5 | ‑ | ‑ | [2] |
| 14 | $n_{p}$ | 2.0 | ‑ | ‑ | [2] |
| 15 | $f_{br}$ | ‑ | 0.28 Hz | Respiratory frequency | Estimated from experimental data |
| 16 | σ(*ζ*) | ‑ | 0.79 Hz | Standard deviation of the respiratory frequency | Estimated from experimental data |
| 17 | *p_hdr_* | ‑ | 0 mm Hg in the supine position and  4.8 mmHg in the upright position | Changes in hydrostatic blood pressure on baroreceptors during the transition from the supine to upright position | Fitted to data |
|  | | | | | |
| **#** | **Parameter** | **Initial value** | **Adjusted value** | **Physiological meaning** | **Source** |
| **The values below were refined during the fitting procedure** | | | | | |
| 18 | $T_{0}$ | 0.5 - 0.7 s | 1.2 s | Time between contractions of the denervated heart | [6] |
| 19 | σ^2^(*ξ*) | 0.0 s^2^ | 0.03 s^2^ | Heart rate variations due to humoral and central factors | Fitted to data |
| 20 | $k_{p}^{B}$ | 1.0 mmHg | 2.0 mmHg | ‑ | Fitted to data |
| 21 | $\hat{s}$ | 70 mmHg | 40 mmHg | ‑ | [2] |
| 22 | $R_{0}$*С* | 2.0 s | 1.5 s | Resting elasticity of blood vessels | [5] |
| 23 | $k_{R}^{v}$ | 0.01 | 0.03 | ‑ | [2] |
| 24 | $k_{1}$ | 0.02 mmHg^-1^ | 0.10 mmHg^-1^ | ‑ | [2] |
| 25 | $p_{0}$ | 40-50 mmHg | 40 mmHg | Minimal pressure that elicits a baroreflectory response | [7] |
| 26 | $k_{2}$ | 0.00125 s·mmHg ^-1^ | 0.005 s·mmHg ^-1^ | ‑ | [2] |
| 27 | $a_{s}$ | -1.65 | -2.50 | ‑ | [8] |
| 28 | $b_{s}$ | 1.0 | 0.5 | ‑ | [8] |
| 29 | $v_{s}^{0}$ | 0.95 | 6.5 | Resting tone of the sympathetic control of heart rate | Fitted to data |
| 30 | $k_{s}^{r}$ | 0.0 | 0.23 | ‑ | Fitted to data |
| 31 | $k_{1}^{l}$ | 0.02 | 0.1 mmHg^-1^ | ‑ | [2] |
| 32 | $p_{0}^{l}$ | 40-50 mmHg | 40 mmHg | Minimal pressure that elicits a baroreflectory response | [7] |
| 33 | $k_{2}^{l}$ | 0.00125 s·mmHg ^-1^ | 0.001 s·mmHg ^-1^ | ‑ | [2] |
| 34 | $a_{s}^{l}$ | -1.65 | -2.50 | ‑ | [8] |
| 35 | $b_{s}^{l}$ | 1.0 | 0.5 | ‑ | [8] |
| 36 | $v_{s}^{l0}$ | 0.95 | 6.5 | Resting tone of the sympathetic control of heart rate | Fitted to data |
| 37 | $y_{s}^{l}$ | 1.0 | 1.0 | ‑ | [2] |
| 38 | $k_{s}^{lr}$ | 0.0 | 0.23 | ‑ | Fitted to data |
| 39 | $v_{p}^{0}$ | 0.0 | 0.0 | Resting tone of the parasympathetic control | [2] |
|  | | | | | |
| **#** | **Parameter** | **Initial value** | **Adjusted value** | **Physiological meaning** | **Source** |
| 40 | $k_{p}^{b}$ | 0.3 | 0.2 | ‑ | [1] |
| 41 | $k_{p}^{r}$ | 0.1 | 0.23 | ‑ | [1] |
| 42 | $k_{c}^{S}$ | 0.7 | 0.034 | ‑ | [2] |
| 43 | $\theta_{c}$ | 1.65 s | 1.5 s | Delay caused by the time that noradreniline needs to produce an effect on heart | [1] |
| 44 | $k_{v}^{S}$ | 0.5 | 0.5 | ‑ | [2] |
| 45 | $\theta_{v}$ | 4.2 | 2.5 s | Delay caused by the time that noradreniline needs to produce an effect on vessels | [2] |
| 46 | $k_{\phi}^{p}$ | 5.8 | 2.0 | ‑ | [2] |
| 47 | $\theta_{p}$ | 0.5 s | 0.25 s | Delay caused by the time that acetylcholin needs to produce an effect on heart rate | [2] |

**References**

1. Seidel H, Herzel H. Bifurcations in a nonlinear model of the baroreceptor-cardiac reflex. Physica D: Nonlinear Phenomena 1998; 115: 145-160.
2. Kotani K, Struzik ZR, Takamasu K, Stanley HE, Yamamoto Y. Model for complex heart rate dynamics in health and disease. Phys Rev E 2005; 72: 041904.
3. Rosenblueth A, Simeone FA. The interrekations of vagal and accelerator effects of the cardiac rate. Am. J. Physiol. 1934; 110: 42-55.
4. Warner HR. The frequency-dependent nature of blood pressure regulation by the carotid sinus studied with an electric analog. Circulation 1958; 6: 35-40.
5. Ringwood JV, Malpas SC. Slow oscillations in blood pressure via a nonlinear feedback model. Am J Physiol Regulatory Integrative Comp Physiol 2000; 280: R1105-R1115.
6. Iaizzo PA. Handbook of Cardiac Anatomy, Physiology, and Devices. p. 187. 2009
7. Taher MF, Cecchini AB, Allen MA, Gobran SR, Gorman RC, Guthrie BL, Lingenfelter KA, Rabbany SY, Rolchigo PM, Melbin J Baroreceptor responses derived from a fundamental concept. Annals of Biomedical Engineering. 1988 ;16(5):429-443.
8. Karavaev AS, Ishbulatov YuM, Borovkova EI, Kulminskiy DD, Khorev VS, Kiselev AR, Ponomarenko VI, Gridnev VI, Prokhov MD, Bezruchko BP Reconstructions of model equations of time-delay system from short experimental time series. International Journal of Modeling, Simulation, and Scientific Computing. 2020; 11(2): 2050014-1.
